# Comparative Mortality of American Cities

**Published:** 1846-08

**Authors:** 


*Comparative mortality of American Cities.*—We are indebted to the New Orleans Medical and Surgical Journal for the data from which is derived the following comparitive view of the mortality of some of the different cities in our country. The plan of bringing their bills of mortality into juxta-position is an excellent one, and especially if it were extended so as to embrace a larger number, with more minute and careful comparison as to the ratio of different diseases, the results would be highly interesting. In connexion with the study of climatic influences, the facts developed in this way would perhaps possess great importance. We have had in view for some time the collection of data for this end, in so far as the bills of mortality in some of the larger and widely separated cities are available. Provided some one who has more leisure for the task does not undertake it, we may perhaps hereafter attempt to carry out our intention. We cannot avoid repeating on this occasion our regrets that our own city is so far behind the intelligence of the age as to make no provisions for registration of deaths and diseases.

*Mortality of New Orleans in* 1845. "The total number of interments in the city of New Orleans for 1845, was 2783; classified as follows viz., White adults, 1287, colored adults, 386; white children, 683; colored children, 381; not distinctly marked, 46.

We have made out the following list of the principal diseases and causes of death: viz, Phthisis, 360; Enteritis, 90; Bilious fever, 20; Congestive fever, 49; typhoid fever, 76; Scarlet fever, 121; pneumonitts, 57, delirium tremens, 73; convulsions, 78; tetanus, 26; trismus nascentium, 109: dysentery, 81; diarrhœa, 56; still-born, 176; old age, 31." The editor attributes the large number of deaths from Phthisis to the fact that a great many go to that place in search of a more genial climate, and there fall victims. He states that the number of cases which originate there is small. The reader will observe the large number of deaths from *trismus nascentium*, which is comparatively unknown in Northern cities, showing pretty conclusively that the disease is directly, or indirectly due to some climatic influences. The results of statistical comparison in this disease suffice, in themselves, to disprove any hypothesis respecting its etiology which does not relate to these influences. The editor explains the large number of deaths from *old age*, in part, by the indefinite use of the term in the reports, some of the ages on the bills being as low as 63. Taking the average annual population of New Orleans at 150,000 the ratio of mortality is about 1 in 54, or within a fraction of 1-85 per cent.

*Mortality of Boston in* 1845. [The following statement is from the Boston Med. and Surg. Journal.] From the general abstract of the Bill of Mortality of the city of Boston just published from the records kept at the health office, it appears that the whole number of deaths during the last year was 1585, being 344 more than during the year previous. Of this number there were still-born, 245: under one year, 481 deaths; under five years 1096; and over 60 years 278. The whole number of deaths from consumption was 426; scarlet fever, 152; lung fever, 35; typhus fever, 97; small pox, 31; delirium tremens, 4.

Taking the population of Boston to be 114,000, the above report shows the mortality to have been 1 in 44,10 or 2,25 pr, ct. With the common ideas (prejudices as these figures show them to be) of the unheathiness of New Orleans, this comparison must appear to most persons surprising. The editor of the New Orleans Journals says, "New Orleans is looked upon, abroad, as a perfect *charnal house*—a *Golgotha*—a "whited sepulcre," fair enough without, but within "filled with dead men's bones;" but the results of careful investigation would lead to a very different opinion. Will it be said that we are fortunate in having one of our *most healthy years* for the comparison? It is true we had no epidemic last summer: but we had as much other sickness as usual, when yellow fever does not prevail. And as for the other seasons of the year, we had about as much sickness as customary. Indeed, we had one epidemic (scarlatina) throughout the year. The fact is, yellow fever is the only disease we have to dread in

New Orleans; and when that does not visit us, no city is blessed with a larger share of health."

We would direct attention to the marked contrast in respect to the deaths from delirum tremens in the two cities. In order to estimate the importance of climatic, or other local influences, it is important to know the plan of treatment which prevails in both places. Will the editors of the Boston and New Orleans Journals enlighten us on this point. The inquiry appears to us one of considerable interest.

*Mortality in Mobile in* 1845.—Number of deaths, 442—of which 320 were whites, and 122 colored. Estimating the population at 15,000, the mortality is about 1 in 34; or 2·94 per cent. The following is a list of the principal diseases, and the number of deaths from each, viz:—Consumption, 57; enteritis, 19; remittent fever, 3; congestive fever, 9; typhus fever, 2; scarlet fever, 1; pneumonia, 19; delirium tremens, 21; convulsions, 23; tetanus, 2; trismus nascentium, 1; dysentery, 11; diarrhoea, 11; still born, 13; old age, 8.

*Mortality of New-York in* 1845. [From the New-York Journal of Medicine.]—Whole number, 10,983; of these were white males, 5,554; white females, 4,892; black males, 261; black females, 276. Five years and under, 4,865; five to ten years, 410; ten to twenty, 389; twenty to thirty, 1,161; thirty to forty, 1,131; forty to fifty, 760; fifty to sixty, 417; sixty to seventy, 343; seventy to eighty, 206; eighty to ninety, 111; ninety to one hundred, 21; one hundred and over, 3; unknown, 106.

The principal causes of death were as follows:—Consumption, 1,659; convulsions, 721; fevers, 501; cholera infantum, 523; apoplexy, 252; congestion of brain, 186; violent deaths, 165; old age, 113.

The population of New-York being taken at 372,000, the mortality is 1 in about 33-89, or 2 95 per cent.

The necessity of some fixed nomenclature in order that the bills of mortality may possess uniformity, is sufficiently apparent on comparing the bills of the different cities. In that of New-York we have no returns under the head of delirium tremens, for example, but how many cases are included under the head of convulsions and congestion of the brain, it is impossible to say. The National Convention appointed a committee to report a nomenclature for this object, which, we trust, will prove satisfactory, and be generally adopted.

*Mortality in Charleston, S. C.* 1845.—Deaths, 563. Under five years, 146; over eighty years, 35. By consumption, 106; dropsy, 45; fevers, 40; teething, 20; tetanus, 13; inflammation, 41. The ratio to population is 1 to 53 1-4.

In the absence of any registration in this place, we give that of a neighboring city, Rochester, in which the records appear to have been made with more particularity than in any of the several cities mentioned. We wish our city might imbibe a spirit of rivalry in this particular, and for this, if for no better motive, imitate her example. Whole number, 502. The population being 25,207, the rate of mortality is 1 to 50-21, or a fraction under 2 per cent. The following is a list of the diseases in 364 of the deaths, the remainder not being embraced in the record:

Inflammation of brain, 4, lungs, 15, bowels, 20, throat, 1, kidneys, 1: fevers—fever, 1, typhus, 7, bilious, 6, childbed, 2, chill, 1, scarlet, 9: disease of brain, 7; congestion of brain, 5; dropsy of brain, 8; disease of heart, 4; enlargement of heart, 1; dropsy of heart, 1; disease of stomach, 1; disease of bowels, 2; ulceration of stomach and bowels, 1; rupture, 1; diarrhœa, 2; dysentery, 1; chronic dysentery, 1; cholera morbus, 1; summer complaint, 26; sore mouth, 1; sore throat, 3; croup, 3; influenza, 4; bronchitis, 3; abscess, 1; abscess of chest, 1; pleurisy, 1; tumor, 1; canker, 2; ruptured bloodvessel, 1; bleeding from lungs, 1; sea sickness, 1; consumption, 77; hooping cough, 4; convulsions, 12; apopletic fits, 2; still born, 25; premature, 2; childbed, 2; accident, 3; drowned, 7; burned and scalded, 6; old age, 4; weakness, 1; insanity, 1; suicide, 1; intemperance, 2; choked, 1; drinking cold water, 1; cancer, 2; erysipelas, 5; dropsy, 3; fever sore, 1; worms, 2; complicated disease, 2; rheumatism, 1; measles, 12; quinsey, 1; liver complaint, 2; chronic calomel disease, 1; diabetes, 1; gravel, 1; spinal affection, 1; swelling of neck, 1; teething, 1; unknown, 24.

